# Microsecond precision of interaural time differences processing in the medial superior olive studied by a computational model

**DOI:** 10.1186/1471-2202-14-S1-P46

**Published:** 2013-07-08

**Authors:** Petr Marsalek, Zbynek Bures

**Affiliations:** 1 Institute of Pathological Physiology, First Medical Faculty, Charles University in Prague, U Nemocnice 5, 128 53, Praha 2, Czech Republic; 2 Faculty of Biomedical Engineering, Czech Technical University in Prague, Nam. Sitna 3105, 272 01, Kladno, Czech Republic; 3 College of Polytechnics, Tolsteho 16, 586 01, Jihlava, Czech Republic

## 

The medial superior olive (MSO) neural circuit in auditory brainstem computes sound azimuth from the interaural time difference (ITD) [1]. High spike timing precision in the order of tens of microseconds is necessary for this neural computation. This makes the MSO an ideal object to study spike time codes, as the relevant information is encoded by the spike timing relative to sound phase.

**Figure 1:**
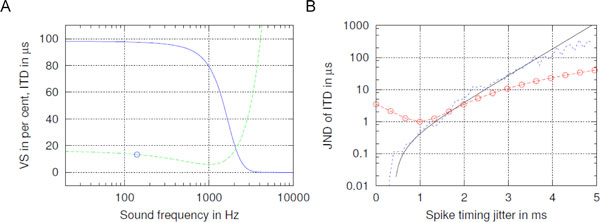
**MSO precision in dependence on sound frequency and spike timing jitter**. (A) Vector strength (VS), in units per cent shown together with the smallest detectable ITD in µs in dependence on sound frequency. Sound frequency 140 Hz used in [2] is shown by circle. (B) Just noticeable difference of interaural time difference depending on variation of the spike timing jitter. Jagged line: simulated data from [3], solid line: an exponential fit to the simulations under the assumption of arbitrary time precision in the model circuit, line with circles: a quadratic function estimate of spike timing precision in a system with realistic noise.

In the MSO, spike timing precision deteriorates towards higher sound frequencies. Experimental recordings of Joris [4] demonstrate this by the vector strength (VS) function (see Figure 1A). To explore the effects of the spike timing jitter on the MSO performance, we have introduced a model of the MSO circuit [2,3]. In our previous work, the model was explored using simulations, giving the value ranges of MSO circuit parameters which are necessary for proper functioning of the MSO in mammals [1]. In the present work we complement the simulations with several fits of smooth functions to the data and with analytical calculations. Figure. 1A shows the shortest ITD detected by the model circuit, with its minimal value at sound frequency 1 kHz. Figure. 1B shows the MSO circuit precision in dependence on the spike timing jitter, which is defined as standard deviation of individual spike times relative to the sound phase. Figure 1B compares the simulated system, jagged line, and exponential curve fitted to the simulation with the analytical estimates of the just noticeable difference (JND) of ITD, line with circles.
